# To determine validity of ultrasound in predicting acute appendicitis among children keeping histopathology as gold standard

**DOI:** 10.1016/j.amsu.2018.11.019

**Published:** 2018-12-18

**Authors:** Ubaidullah khan, Murad Kitar, Imed Krichen, Kais Maazoun, Rasha Ali Althobaiti, Mostafa Khalif, Mohammad Adwani

**Affiliations:** Pediatric Surgery, Department of Surgery, Alhada Armed Forces Hospital, Taif, Saudi Arabia

**Keywords:** Acute appendicitis, Ultrasound, Histopathology

## Abstract

**Background:**

To determined the accuracy of ultrasound in diagnosis of acute appendicitis in children keeping histopathology as gold standard.

**Methods:**

A prospective evaluations of all ultrasound for appendicitis from January 1, 2014, to June 15, 2017, was conducted at our hospital. A diagnostic protocol was implemented to reduce radiation exposure employing US as the initial imaging modality followed by CT for non-diagnostic US studies in patients with an equivocal clinical presentation. The imaging, operative findings, and pathology of 223 patients (females 80, males 143, age less than 14years) with diagnosed appendicitis were collected. The sensitivity, specificity, predictive value, and negative appendectomy rate were also analyzed. All those patients which had subjected to surgery were included to evaluate the true result of ultrasound in diagnosis of appendicitis.

**Results:**

Of the 223 pediatric appendectomies performed in this time period, a total of 192 (86%) were diagnosed by ultrasound. The histopathology of 8 was normal (3.6%), CT done in 11 and three was normal. The negative appendectomy rate was 3.6%. US were the sole imaging modality in all patients.

**Conclusions:**

In the diagnosis of acute appendicitis in children, ultrasound is useful and accurate mode, which results in a significant decrease in negative appendectomies with no increase in the number of CT scans. This has important implications in the reduction of childhood radiation exposure.

**Study design:**

cross sectional validation.

## Highlights

• Total 223 Diagnose as acute appendicitis on initial clinical basis and follow by ultrasound study.
• All went for surgery, 8 patient negative by histopathology.
• Diagnosis by ultrasound 192 out of 223, Sensitivity 86%.
• Accuracy reaches 92% with secondary signs on ultrasound finding.

## 1 Introduction

Zero radiation to children, quick, more efficient and easy excess make ultrasound one of leading choice of diagnosis in appendicitis as compared to CT or MRI [1]. Acute abdominal pain is a common cause for surgical consultation in pediatric gastroenterology emergency [2,3]. However, a third of children presenting with acute abdominal pain diagnosed as appendicitis [4]. The diagnosis of acute appendicitis is difficult task, with a considerable proportion of diagnostic errors based on the clinical and laboratory data, this is due to the fact that appendicitis may present under several forms (simple, complicated, mass, and abscess), be diagnosed in several ways (physical examination, laboratory tests, and imaging studies).

The symptoms and clinical signs are not uniform, which then depend on diagnostic imaging for more precise diagnosis. Ultrasound is regarded, by the American College of Radiology (ACR), as the method best suited for initially imaging a patient with suspected acute appendicitis [5]. In contrast, in 2010, the Dutch Society of Surgeons introduced guidelines that recommended the routine use of ultrasound and/or computed tomography (CT) for the diagnosis of acute appendicitis [6].

Preetam G et al. mentions in his talks that US not limited to operator dependency/skill level and patient-specific factors like pain, bowel gas and body mass index (BMI) can be a challenge [7]. The wide range of reported sensitivity and specificity in the ultrasound diagnosis of acute appendicitis in children appears to be because of operator and patient factors [2,4]. In our region and generally, Obesity has been cited as a factor responsible for a significant decrease in the effectiveness of ultrasound [8]. The objective of our study was to prospectively evaluate the progress in diagnosing acute appendicitis with the help of ultrasound keeping histopathology as gold standard.

## 2 Materials and methods

### 2.1 Design and study population

We obtained approval by the hospital Ethics Committee. All patients gave their informed consent for any procedure to be done. Between January 2014 and June 2017, we collected prospectively the demographic data and results of ultrasound examinations performed for acute appendicitis and then follow with histopathology report. All patients had the same general radiological scanning protocol, including ultrasound and CT scans in difficult cases. All patients were less than or equal to 14 years of age and data were collected of all patients for acute appendicitis either from the hospital emergency department or from inpatient. The study has been written in compliance with STROCSS criteria [9].

### 2.2 Sonographic study

An initial ultrasound was performed by one of the senior technicians in diagnostic and interventional imaging. This examination included the entire abdomen and pelvis. Immediately after the technician, a second look of images was carried out by a department doctor, in some cases they assist to scan and pediatric surgery doctor also was present on time of scanning.

#### 2.2.1 Diagnostic categorization

**Fig. 1:**
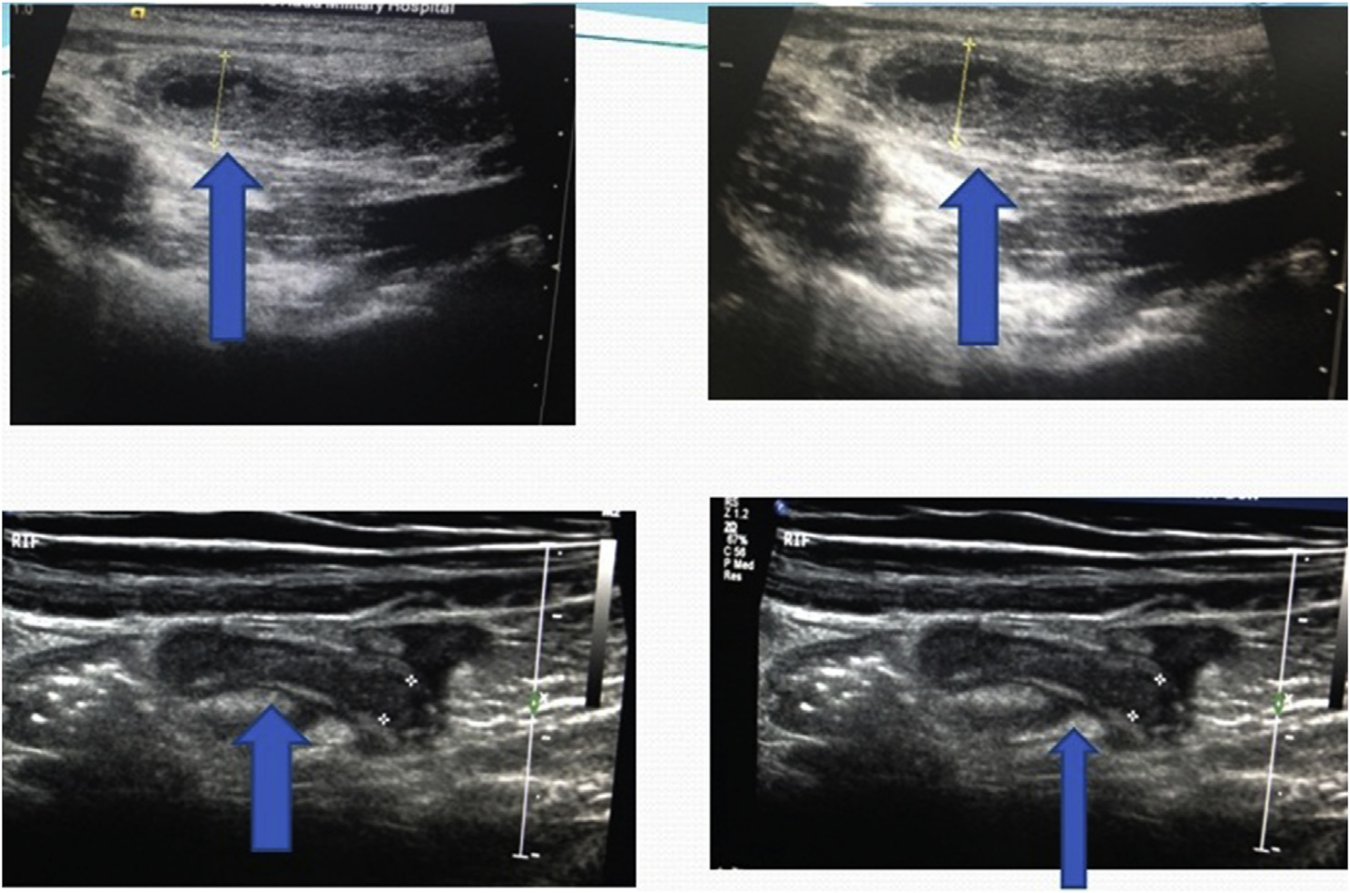
Ultrasonography images of appendix with arrow marks.

The ultrasound scanning included the visualization of the appendix (non-visualization, complete visualization), the transverse diameter of the appendix (less than 6 mm, between 6 and 8 mm, greater than 8 mm); the wall's differentiation preserved or not [Fig. 1]. Other finding to look for was: Localized tenderness, guarding and increase vascular flow, regional mesenteric lymphadenopathy, and free intraperitoneal fluid or collection and fecolith. We also pick complications such as a mass or abscess. At the end of the procedure, following diagnostic conclusions were possible: acute appendicitis, likely appendicitis and normal appendix.

Acute appendicitis was diagnosed when an appendix was enlarged, wall differentiation was not preserved, associated localized tenderness was seen, secondary signs (hyperemia, free fluid or collection) was observed, increase diameter and fecolith seen. A normal appendix was diagnosed when a normal-sized appendix was observed and no signs were seen. In case of non-visualization of the appendix, all appendicitis signs were absent then further decision was taken on clinical grounds and CT scan was choice of investigation in equivocal cases as in severe diarrhea or gastroenteritis.

### 2.3 Ultrasound analysis

The surgeon and radiologist agree upon the final ultrasound diagnosis was: acute appendicitis = A, probable appendicitis = B and normal appendix = C.

### 2.4 Diagnostic errors

An imaging result was considered false positive when the final ultrasound report was acute appendicitis (A) or probable appendicitis (B) but the appendix was healthy at surgery, a complementary examination such as computed tomography (CT) was unremarkable. A result was considered false negative when the final sonographic classification was normal appendix or appendicitis unlikely, but a diagnosis of acute appendicitis was established by pathological means.

### 2.5 Computed tomography (CT) examination

CT was done in selected and difficult cases, where US was normal or not visualized appendix in contrast to clinical examination mimic appendicitis verses gastroenteritis.

### 2.6 Histopathological analysis

It was our gold standard to compare with US finding. Negative appendectomy was defined as, an operation with a preoperative diagnosis of appendicitis, and absence or minimal acute inflammatory cells in the case of appendectomy, or normal appearance of the appendix. The existence of polymorphonuclear leukocytes, lymphocytes, or plasma cells in appendiceal biopsy was considered positive for appendicitis.

### 2.7 Descriptive statistics

We calculated the sensitivity, specificity and positive predictive value. These calculations were made over the course of study period and during the first and the second year periods difficulties encountered. The gold standard was defined as the pathological diagnosis for the patients treated surgically. Statistical analyses were performed using the SPSS 20.

## 3 Results

### 3.1 Population

All children (223) with acute appendicitis agreed to be in the study and had an ultrasonography examination follow by appendectomies. During the study period, 223 patients were included: 80 (35.9%) girls and 143 (64.1%) boys. The median age was 9.9 years, with a range of 3–14 years. The mean weight for both genders was 31.5 kg; the median weight was 32 kg, with extremes of 13 and 64 kg.

### 3.2 Ultrasonography

#### 3.2.1 Ultrasound visualization of the appendix

**Fig. 2:**
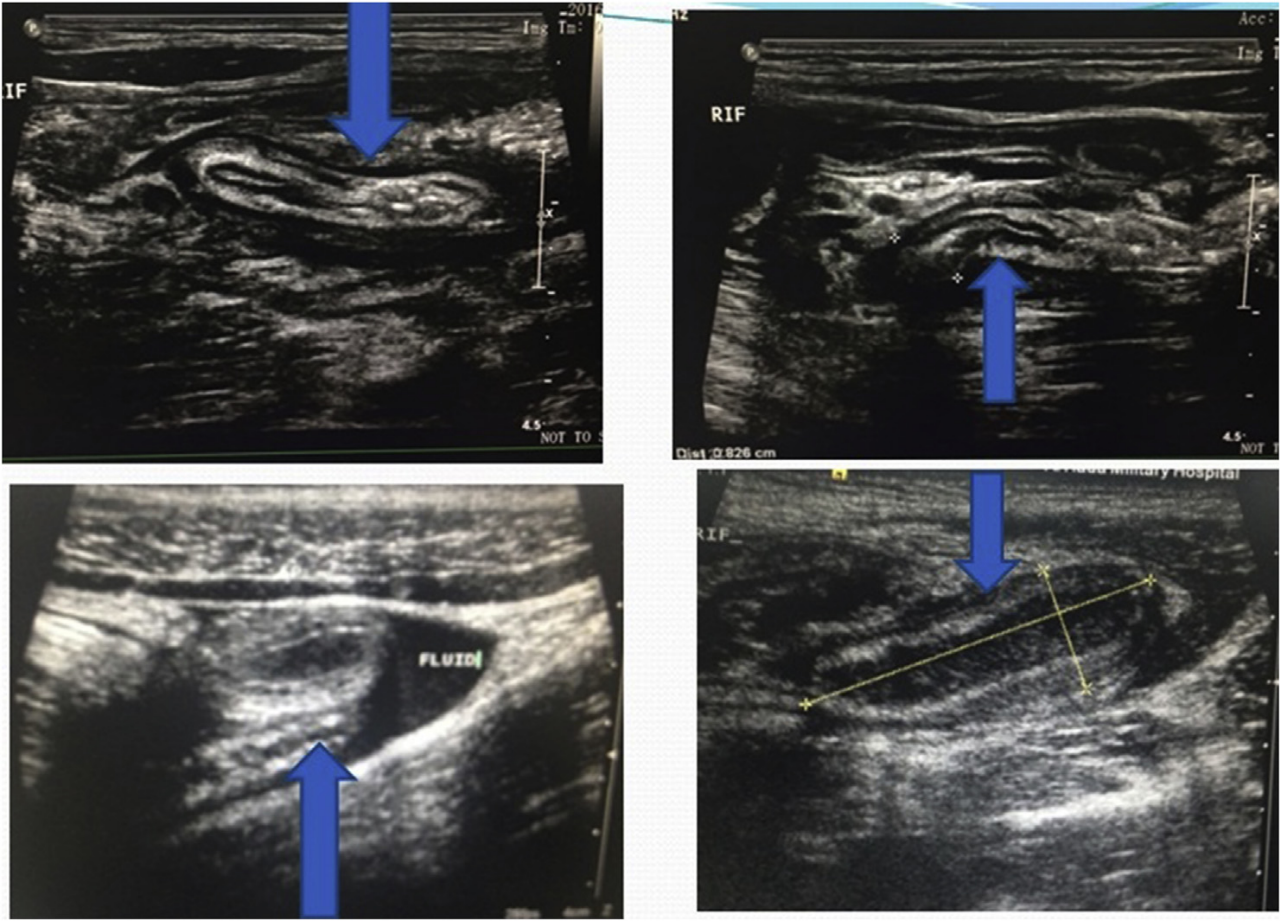
Transverse/Longitudinal linear gray-scale US images of acute appendicitis (arrows).

The ultrasonographer visualized the appendix in 192 of the 223 patients (86%) and secondary signs in 14(6%) [Fig. 1, Fig. 2 ]. The appendix was not identified in 17patients (8%). The secondary signs were hyperemia, fecalith, free fluid or collection. The proportions of completely visualized appendices with secondary signs were significantly higher [Fig. 1, Fig. 2, Fig. 4].

#### 3.2.2 Inflamed appendix

**Fig. 3:**
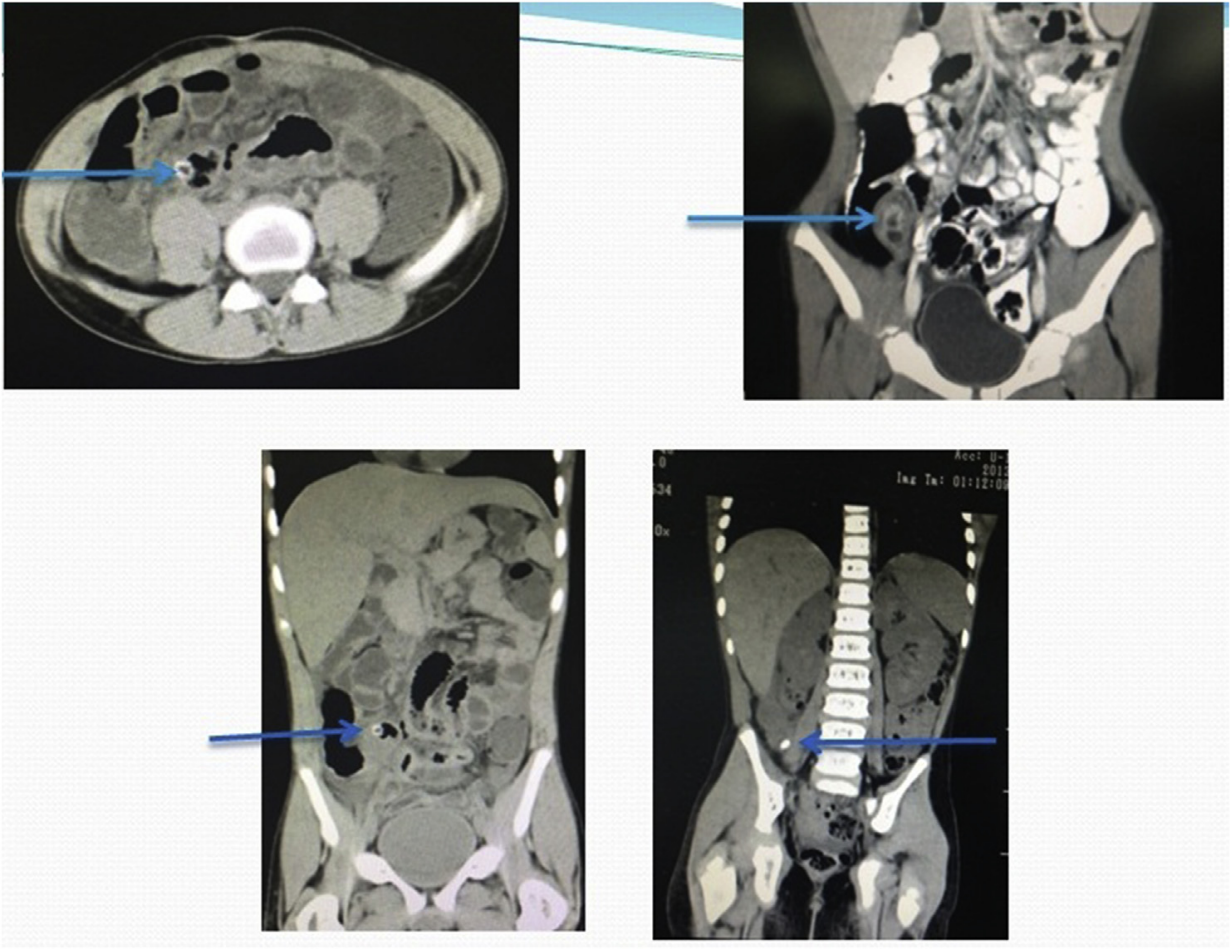
CT images show fecalith and acute appendicitis in equivocal cases (arrows).

Of the 223 patients studied, 215 had appendicitis, 8 of which were normal and all confirmed by pathological analysis. 13 have diagnosed as complicated appendicitis (9 perforations and 4 mass) confirmed by radiological, surgical and pathological results. In 17 cases, appendix was not identified during ultrasound examination and CT scan was performed in 11, out of which 8 was suggestive of appendicitis. The 3 CT scans were interpreted as normal (one patient re-scan by ultrasound diagnosed as inflamed appendicitis) [Fig. 3]. The other 6 patients were operated on clinical basis as suspected acute appendicitis. Surgeons have decided to operate depending mainly on the clinical and ultrasonographical data. Of the 223 patients 192 (86%) with a definite diagnosis of acute appendicitis if including those with presence of secondary signs 14(6%) the number reach to 206 (92%). 31 (14%) were considered as negative by the ultrasound (false negative) [Fig. 5]. However, 8 false-positive diagnoses (3.6%) miss or not seen by ultrasound resulted in a negative appendectomy turn out to be healthy appendix on histopathology; had been operated and diagnosed by the clinical data as presume to be having appendicitis.

**Fig. 4:**
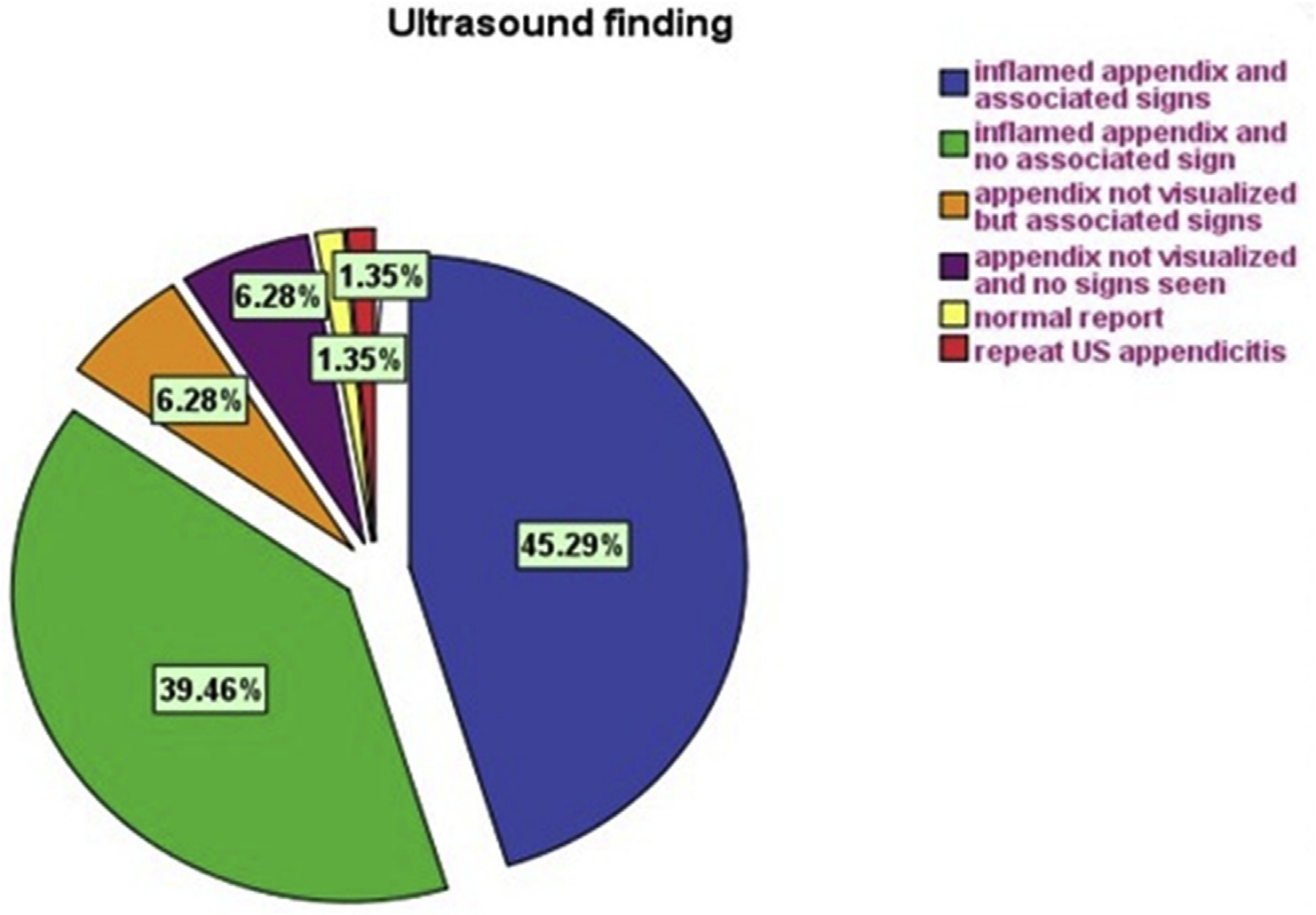
Ultrasound finding of 223 patients.

**Fig. 5:**
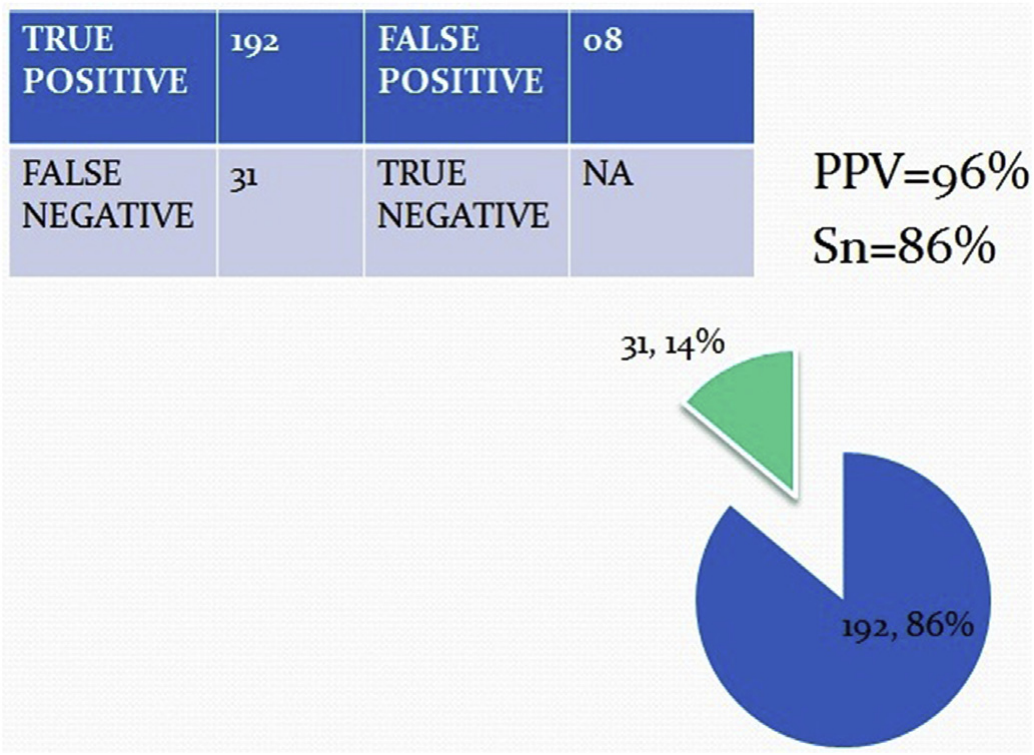
Sensitivity and specificity of US.

#### 3.2.3 Degree of diagnostic certainty

**Fig. 6:**
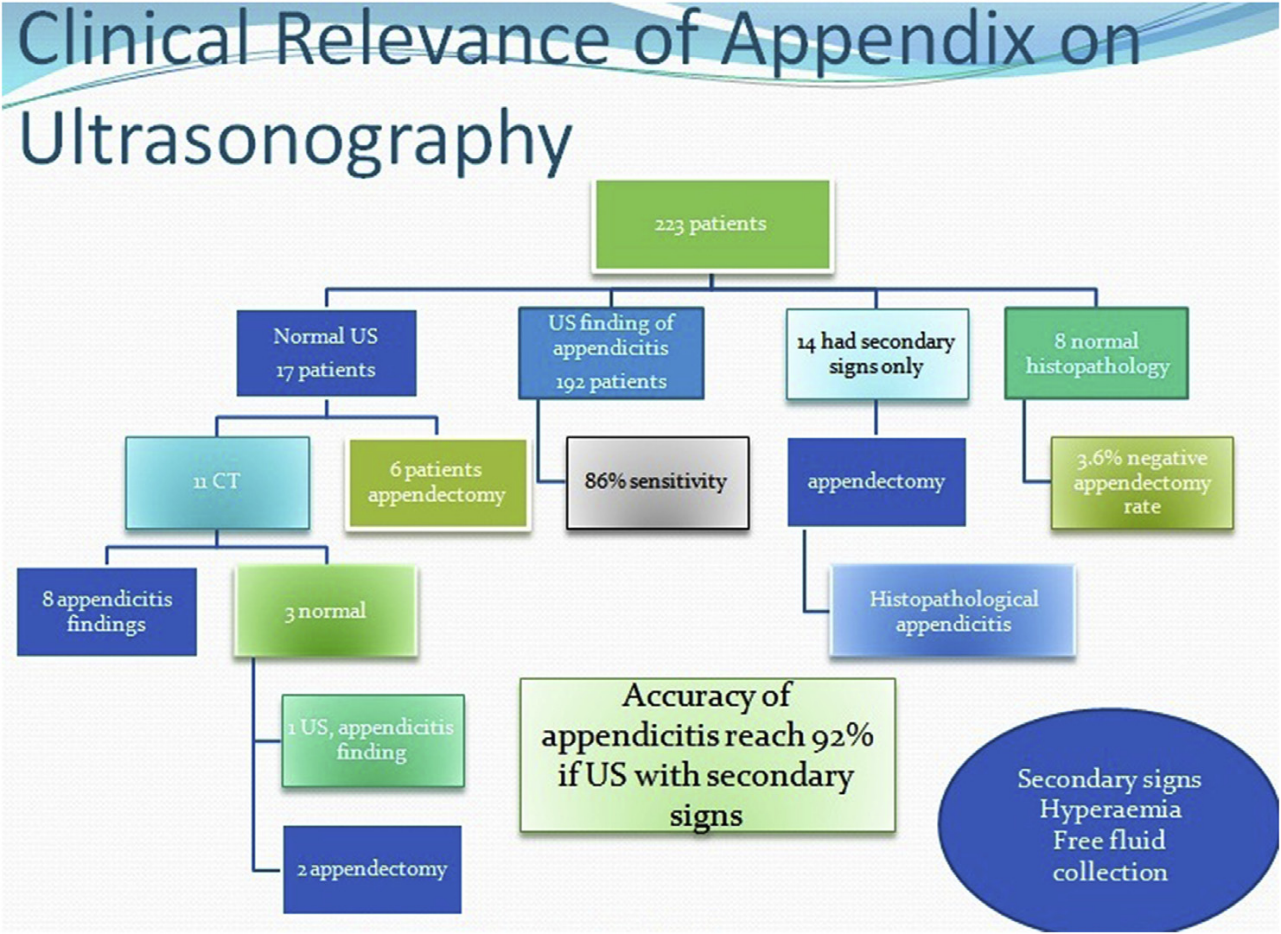
Flow chart showing final outcome of US study.

At the end, one of possible diagnoses was chosen: acute appendicitis (A), probable appendicitis (B) and normal appendix (C). In those cases where the diagnoses confirmed by radiological scanning followed by surgeon and histopathology confirmation for acute appendicitis (A) or normal appendix (C), the diagnoses were considered correct. When the appendix was completely visualized, the degree of confidence was 86% by ultrasound and 92% with inclusive of secondary signs [Fig. 6].

## 4 Discussion

Our results showed good diagnostic performance by the ultrasound during the study period [Fig. 5, Fig. 6]. The values for sensitivity (0.86), specificity (0.97), and accuracy (0.92) we obtained for the ultrasound are similar to those reported in meta-analyses [1,4,[10], [11], [12], [13]]. We also observed no statistically significant difference between the diagnostic performance achieved by the technicians and that of doctors. However, morning results were better [14]. It emphasizes the capacity work load of a surgeon with radiology to be efficient in the ultrasound diagnosis of appendicitis and a progression of their ability during their work as a team, as our technical staff in the majority of cases. The false positive cases of appendicitis were misdiagnosed because of limitation such as obesity, operative dependent and associated medical issue like gastroenteritis where CT scan was not even helpful in three of our cases. Andrea S. at el have meta-analysis and found no statistically significant differences in the sensitivity, specificity, or diagnostic odds ratio of second-line US, CT, or MRI in children or adults [13].

We find ultrasound as good first imaging procedure for pediatric appendicitis, with pathological-surgical correlation. Dalia M et al. also show decrease CT usage and US as more promising image modality [15]. Andreana B et al. in their retrospective analysis found average size 6.93 although sample size was small, But, in our observation upper normal value of 6 mm is probably too sensitive [7,16,17]. Among these 31 patients, the appendix was either not visualized at all (17 patients) or with secondary signs (14 patients), by the radiologist in all cases, emphasizing the importance of visualization of the appendix.

Tristan R et al. saw appendicitis out of 230 number in 68.7%cases, mark improvement with negative appendectomy rate of 8.7% and with secondary signs increase sensitivity [12,18]. The rate of visualization of the appendix by ultrasound has varied widely in the literature. Lee et al. recorded a rate of visualization of the appendix of 99% among patients in all age groups [7,12,19]. As in our study, visualization was more frequent in acute appendicitis (86%) than when the appendix was healthy (3.6%). Mittal et al. have found a lower rate of visualization of the appendix in the hospitals where US is used less often (25%) compared to hospitals where ultrasound is always available (56%) [2].

Taken into consideration weak side of US, dependent on operator, improving with help of new technology and being as a surgeon role to assist in cases bring the mystery of diagnosis to the end [Fig. 6]. With all this bunch of information ahead of surgery for confirmation that it is appendicitis, like signs/symptoms (pain and vomiting) which we notice, then clinical examination, blood test like CRP(c-reactive protein) in our patients high significance and last imaging study(US/CT) which proved to be diagnostic. Depend on policy of your hospital as in our setup US scan is for every suspected case of appendicitis (24hr available), trained staff as our technical operator which are more efficient in visualization of appendix and then the surgeon job to help along during whole process. Similarly, authors have shown that specialized pediatric sonographers identified the appendix more often than the general sonographer [1,20,21].

In contrast, in our study rates of visualization of the appendix were higher that way much comparable to other studies. Shireen A et al. concluded that nonvisualized appendix on ultrasound imaging and no evidence of secondary inflammatory changes, the likelihood of appendicitis is less than 2% [11]. Secondary signs may also improve the diagnostic accuracy of equivocal ultrasounds for suspected appendicitis in children, Peter C et al. stated that ultrasound for diagnosing complicated appendicitis or an appendicolith, the high specificity and NPV suggest that ultrasound is a reliable test [22].

The National Cancer Institute and the American Pediatric Surgical Association recommend use of non-radiation based imaging such as US where possible [23]. Currently, over 50% of children undergoing appendectomy in North America have radiation based imaging [24]. This rate is too high and a tailored approach based on risk is sensible, especially in children. Universal imaging of patients with CT, apart from consuming resources, is not without health risks. It has been estimated that the benefit of universal imaging in avoiding 12 unnecessary appendectomies could result in one additional cancer death [25].

## 5 Conclusion

TAKE-HOME MESSAGE from Mark J. Favot and Robert R. Ehrman, was given in their paper as follow: In children with suspected appendicitis, history, physical examination, laboratory results, and the Pediatric Appendicitis Score cannot safely rule in or rule out the diagnosis. A positive ultrasonographic result, with sonography performed by a qualified provider, is diagnostic [26].

The ultrasound evaluation of suspected acute appendicitis in children, performed by radiology technician and doctor, in 92% of cases accurate in our study, confirm by histopathology [Fig. 6]. Ultrasound as first-line imaging for suspected pediatric appendicitis and prompt radiological-surgical correlation could help to improve diagnostic performance. The usage of CT scan is dramatically decline from start till end in our study period.

## Ethical approval

Approve by hospital ethical board.

## Funding

No funding for this study

## Author contribution

Ubaidullah khan.

Mourad kitar.

Rasha ali althobaiti.

Imed krichene.

Mostafa youssef lotfy khalif.

Maazoun kais.

Mohammad adwani.

## Conflicts of interest

No interest.

## Research registration number

Researchregistry3713.

## Guarantor

The Guarantor is the one or more people who accept full responsibility for the work and/or the conduct of the study, had access to the data, and controlled the decision to publish.

## Type of study

Cross sectional validation.

A prospective collection of data of ultrasound study, done for diagnosis of acute appendicitis.
